# Bending of Thin Liquid Crystal Elastomer under Irradiation of Visible Light: Finsler Geometry Modeling

**DOI:** 10.3390/polym10070757

**Published:** 2018-07-09

**Authors:** Hiroshi Koibuchi

**Affiliations:** Department of Industrial Engineering, National Institute of Technology, Ibaraki College, Nakane 866, Hitachinaka, Ibaraki 312-8508, Japan; koibuchih@gmail.com; Tel.: +81-29-275-3910

**Keywords:** liquid crystal elastomer, light irradiation, dye-doped, Monte Carlo, statistical mechanics, Finsler geometry

## Abstract

In this paper, we show that the 3D Finsler geometry (FG) modeling technique successfully explains a reported experimental result: a thin liquid crystal elastomer (LCE) disk floating on the water surface deforms under light irradiation. In the reported experiment, the upper surface is illuminated by a light spot, and the nematic ordering of directors is influenced, but the nematic ordering remains unchanged on the lower surface contacting the water. This inhomogeneity of the director orientation on/inside the LCE is considered as the origin of the shape change that drives the disk on the water in the direction opposite the movement of the light spot. However, the mechanism of the shape change is still insufficiently understood because to date, the positional variable for the polymer has not been directly included in the interaction energy of the models for this system. We find that this shape change of the disk can be reproduced using the FG model. In this FG model, the interaction between σ, which represents the director field corresponding to the directional degrees of LC, and the polymer position is introduced via the Finsler metric. This interaction, which is a direct consequence of the geometry deformation, provides a good description of the shape deformation of the LCE disk under light irradiation.

## 1. Introduction

Liquid crystal elastomer (LCE) is a material in which LC molecules are chemically bonded to polymers, and its most remarkable property is that the anisotropy in the direction of nematic ordering of the LC molecules is reflected in the macroscopic shape [1,2,3,4,5]. Photoinduced bending of liquid-crystalline gel, which includes azobenzen monomers, shares the same mechanism [6,7]. In other words, the macroscopic shape of the LCE is sensitive to the microscopic orientation of the LCs and vice versa. Indeed, LCE elongates into a spontaneously chosen direction at low temperature if no constraint is imposed on the LC molecules, while at high temperatures, the elongation is suppressed due to the phase change from nematic to isotropic. External fields such as electric field and mechanical stresses also elongate the LCEs, and these field-driven elongations have been studied intensively [8,9,10,11,12].

Because of these interesting properties, a great deal of attention has been paid to LCEs, and studies of their technological applications, such as artificial muscles, have been conducted [13,14,15]. A polymer-dispersed LCE has recently been proposed as a functional and shape-programmable material for additive manufacturing [16]. A cellulose liquid crystal motor has also been proposed [17]. In the statistical mechanical studies of LCEs, the variable σ for directors and strains are introduced, and the obtained results successfully describe the experimental data [2,3,4,5]. However, the strains themselves are not always identical to the LCE shape, so the position vector is better or more straightforward for the analyses of material shape such as polymers [18,19].

In Refs. [20,21], the interaction between the LC molecules and polymers is coarse-grained and implemented in the 3D Finsler geometry (FG) model, and the elongation phenomenon is successfully explained. The soft elasticity, in which the stress-strain diagram includes a plateau, is also reproduced by the FG model. This soft elasticity arises because the director orientation and the shape of material interact with each other. The so-called J-shaped stress-strain diagrams of soft biological tissues such as animal skin and muscles are also reproduced by the 2D FG model [22]. In the FG models for the soft-elasticity and J-shaped diagram, we discard the detailed information of the interaction between the LCs and polymers, and instead, we modify the underlying geometry such that the internal metric function of the materials is changed from Euclidean to Finsler. In this sense, the interaction is determined in a more basic and constructive manner from the geometries inside the material and the space ${ℜ}^{3}$. As a result of this procedure, the interaction between the direction σ of the directors and the position $r(\in {ℜ}^{3})$ of the polymers is automatically introduced in the model. This is in sharp contrast to the ordinary modeling techniques, in which an interaction energy is explicitly introduced in the Hamiltonian. Therefore, this FG modeling technique is completely new and should hence be checked thoroughly by application it to a variety of experimentally observed phenomena.

In this paper, we apply the 3D FG model to the interesting experimental observation that a thin and small LCE disk moves on the water due to light irradiation [1]. This phenomenon has attracted a great deal of attention in engineering applications because it is considered a model for a micro robot actuated by a non-mechanical stimulus [6,7,17]. The driving force that moves the disk on the water comes from the shape change. By moving a spot irradiated by light from the center to the radial direction on the disk surface, the position of the bending on the edge of the disk can be controlled. This shape change at the edge moves in the same direction as the spot; at the same time, the water is pushed in the same direction that the bending edge moves, and then, by the action-reaction principle, the disk moves back or swims in the opposite direction on the water. We expect that such a shape change can be studied by using the FG model because the position variable r is directly included as a variable. Therefore, it is worthwhile to study whether the experimentally reported shape change of the disk is consistent with the results of the 3D FG model.

We should comment that the FG modeling technique can also be applied to a deformation of LCE piece in more general situation even for composite materials such as polymer-dispersed LCE [16]. Such an applicability of FG model is naturally expected because the deformation of the variable r is automatically determined from the variable σ, and the deformation of r is independent of the reason for distorting σ because of the implemented interaction between r and σ.

## 2. Reported Experiment

We describe the experiment reported in Ref. [1] and summarize the current understanding of the shape deformation by light irradiation. A dye-doped nematic LCE is used in the experiment; the disk size is 5 mm in diameter and 0.32 mm thick, so the ratio D/H of thickness *H* and diameter *D* is approximately D/H=16. This small piece of LCE floats nearly motionless on the surface of a water reservoir, which has a depth of 2 cm. The direction of the nematic director, which is the constituent LC molecule, is parallel to the surface.

**Figure 1:**
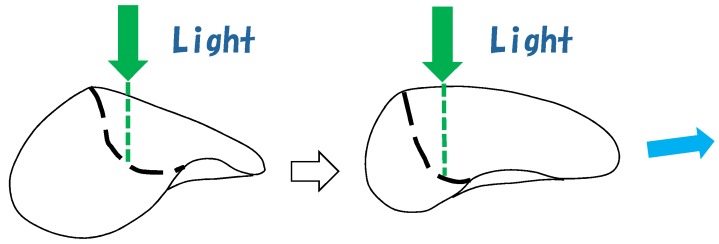
Shape deformation of LCE sample floating on the water under laser light illumination from above [1]. The deformation of the sample edge moves with the movement of the light spot, and at the same time, the LCE sample moves in the opposite direction. The nematic director direction is parallel to the dashed line on the sample surface.

This LCE sample is illuminated from above by an argon-ion laser with a beam width of 3 mm. The light spot is located on the center of the disk in the beginning and is then moved toward an edge along the direction perpendicular to the LC alignment direction. Then, the sample moves opposite to the direction in which the light spot moves (see Figure 1).

The authors of Ref. [1] discussed why the LCE sample moves on the water surface. One possible reason that they suggested was the shape deformation, as shown in Figure 1. A part of sample edge is lifted and strongly deformed compared to the other part of the edge, and this deformed position moves with the movement of the light spot. This deformed edge pushes water in the same direction as the light movement, and by the action-reaction principle, the LCE sample moves in the opposite direction. This is a rough outline of the mechanism of the swimming mentioned in the Introduction.

The effect of light on the dye-doped nematic LCE samples is also discussed in Ref. [1]. Based on these discussions, there are two possible effects: the first is dye-mediated heating due to the light absorption, with the increase in temperature causing a transition from the nematic ordered phase to the disordered or isotropic phase. The second also reduces the orientation order due to the effect of the photoisomerization of the dissolved dye. The dye-doped LC molecules play the role of an impurity if they change from the trans state to the cis state via irradiation. These two effects are expected to reduce the orientation ordering of directors, and this change in the director orientation leads to a shape change. We should note that these two effects have been throughly investigated and are now well understood [23,24,25,26,27,28].

However, it is unclear why a change in the orientation order causes shape deformation. This is actually very difficult to study from the microscopic perspective because the interaction between the LCs and polymers is unclear. Here, “unclear” means that the corresponding Hamiltonian in the statistical mechanical models remains unknown because the polymer position r is not directly used. One of the reasons for the lack of a Hamiltonian that includes r is that the interaction is very difficult to assume due to the hierarchical structure of polymer, which is composed of monomers and forms a crystalline/non-crystalline structure, with these structures also forming domains. All of these hierarchical structures are connected to the shape deformation; for this reason, a simple interaction energy is not expected as the Hamiltonian [29,30,31].

## 3. 3D FG Model of LCE

To understand why a dye-doped nematic LCE deforms under light illumination, we employ a model that is completely different from the models utilized in previous statistical mechanical studies of materials, as mentioned in the Introduction. The model in this paper is an FG model and is identical to the model used in Ref. [21]; however, we describe the model for the readers’ convenience.

**Figure 2:**
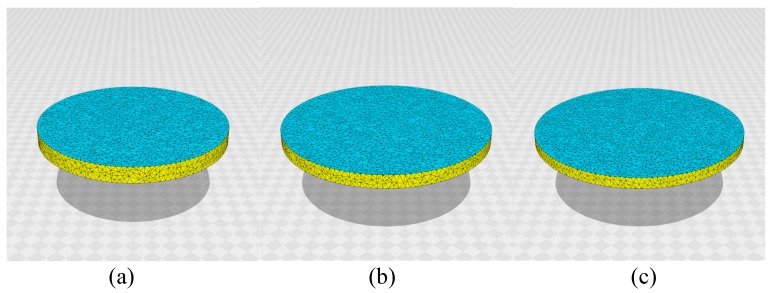
3D disks of three different ratios D/H with diameter *D* and thickness *H*. The ratio D/H and the total number of vertices *N* are (**a**) D/H=12, N= 10,566, (**b**) D/H=16, N= 10,980, and (**c**) D/H=20, N= 10,563. The scales of the figures are the same.

First, the 3D lattices for the simulations are shown in Figure 2a–c. For the LCE sample in the experiment, the ratio D/H of height *H* and diameter *D* is D/H=16, as mentioned in the previous section. Thus, we use three different lattices of D/H=12, D/H=16, and D/H=20, as shown in Figure 2a–c.

We show the data for the lattices, including the lattice of N= 10,346, D/H=8, in Table 1. The size of the lattice is given by $\left(N,N_{B},N_{T},N_{\mathrm{tet}}\right)$, where *N*, $N_{B}$, $N_{T}$, and $N_{\mathrm{tet}}$ are the total numbers of vertices, edges (or bonds), triangles, and tetrahedrons, respectively. The Euler numbers $N-N_{B}+N_{T}-N_{\mathrm{tet}}\left(=1\right)$ of these lattices are identical to those of a tetrahedron, which is characterized by $\left(N,N_{B},N_{T},N_{\mathrm{tet}}\right)=\left(4,6,4,1\right)$, because the cylinders are topologically identical to a tetrahedron. The symbols $N^{U},N^{L}$ denote the total number of vertices on the upper and lower surfaces, respectively, and $N_{B}^{U},N_{B}^{L}$ and $N_{T}^{U},N_{T}^{L}$ respectively denote the total number of bonds and the total number of triangles on these two-dimensional surfaces. These data also satisfy $N^{U,L}-N_{B}^{U,L}+N_{T}^{U,L}=1$ because a disk is topologically identical to a sphere with a hole, e.g., a triangle.

To describe the model, we start with the continuous Gaussian bond potential such that

(1) $$\begin{array}{cc} S_{1}\left(r,\sigma \right) & =\int \sqrt{g}d^{2}xg^{ab}\frac{\partial r}{\partial x_{a}}\cdot \frac{\partial r}{\partial x_{b}}, \end{array}$$

where $r(\in {ℜ}^{3})$ is a material position, $x_{a}\left(a=1,2,3\right)$ is a local coordinate inside the material, *g* is the determinant of the Finsler metric $g_{ab}$, which is a 3×3 matrix, and $g^{ab}$ is the inverse of $g_{ab}$. This continuous $S_{1}$ is discretized on 3D lattices such as those in Figure 2a–c, which are composed of tetrahedra. Please note that this $S_{1}$ depends not only on r but also on $\sigma (\in S^{2}:\mathrm{unit}\;\mathrm{sphere})$, which is a director field corresponding to the directional degrees of freedom of the LC molecule. The dependence of $S_{1}$ on σ comes from the fact that $g_{ab}$ depends on σ. Note also that this $S_{1}$ is a 3D extension of 2D $S_{1}$, which is a model for the membranes [32,33,34,35,36,37,38], and 2D $S_{1}$ is an extension of the 1D model for polymers [39]. Therefore, this type of Gaussian potential $S_{1}$ in Equation (1) is of fundamental importance in the studies of polymers.

**Figure 3:**
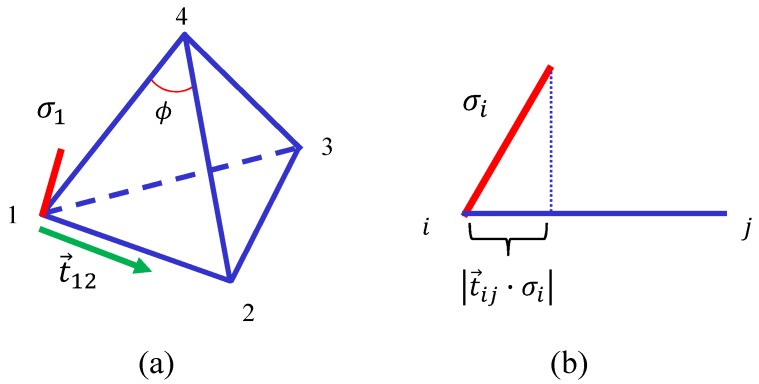
(**a**) A tetrahedron, which forms lattices for the simulation, and a variable $\sigma_{1}$ at the vertex 1, and (**b**) the tangential component $|t_{ij}\cdot \sigma_{i}|$ of $\sigma_{i}$ along the bond ij.

To evaluate the dependence of $S_{1}$ on σ, we write the discrete 3D Finsler metric $g_{ab}=\left(\begin{array}{ccc} 1/v_{12}^{2} & \;0 & \;0\\ 0 & \;1/v_{13}^{2} & \;0\\ 0 & \;0 & \;1/v_{14}^{2} \end{array}\right),$ which is obtained by replacing the element 1 of Euclidean metric $\delta_{ab}$ with $1/v_{ij}^{2}$. This Finsler metric is defined on the tetrahedron in Figure 3a, where the local coordinate origin is at the vertex 1. In this $g_{ab}$, $v_{ij}$ is given by

(2) $$\begin{array}{c} v_{ij}=\left|t_{ij}\cdot \sigma_{i}\right|+v_{0},\;t_{ij}={\vec{\ell }}_{ij}/\ell_{ij},\;{\vec{\ell }}_{ij}=r_{j}-r_{i}, \end{array}$$

where $t_{ij}$ is the unit tangential vector from the vertex *i* to *j* (Figure 3b). This $v_{ij}$ corresponds to the unit of the Finsler length [20,40,41,42], and it depends on σ; hence, $g_{ab}$ depends on σ. We should note that $v_{0}$ in $v_{ij}$ plays the role of a cut-off because $1/v_{ij}^{2}$ is divergent if $t_{ij}$ is vertical to $\sigma_{i}$. We fix $v_{0}$ to $v_{0}=0.001$ in this paper.

A discrete version of $S_{1}$ is obtained by replacing the differentials $\partial_{a}r$ with differences such as $r_{j}-r_{i}$ and the integral $\int d^{2}x$ with the sum $\sum_{i}$. Including several terms, we have the Hamiltonian S(r,σ) such that

(3) $$\begin{array}{c} \begin{array}{c} S\left(r,\sigma \right)=\lambda S_{0}\left(\sigma \right)+S_{1}\left(r,\sigma \right)+\kappa S_{2}\left(r\right)+U_{3D}\left(r\right)+U_{\mathrm{vol}}\left(r\right),\\ S_{0}\left(\sigma \right)=\frac{1}{2}\sum_{ij}\left(1-3{\left(\sigma_{i}\cdot \sigma_{j}\right)}^{2}\right),\\ S_{1}\left(r,\sigma \right)=\sum_{ij}\Gamma_{ij}\ell_{ij}^{2},\;\Gamma_{ij}=\frac{1}{4\bar{N}}\sum_{\mathrm{tet}}\gamma_{ij}\left(\mathrm{tet}\right),\;\ell_{ij}^{2}={\left(r_{i}-r_{j}\right)}^{2},\\ S_{2}\left(r\right)=\sum_{i}\left[1-\cos(\phi_{i}-\pi /3)\right],\\ U_{3D}\left(r\right)=\sum_{\mathrm{tet}}U_{3D}\left(\mathrm{tet}\right),\;U_{3D}\left(\mathrm{tet}\right)=\left\{\begin{array}{cc} 0 & \left(\mathrm{Vol}(\mathrm{tet})>0\right)\\ \infty & \left(\mathrm{otherwise}\right) \end{array}\right.,\\ U_{\mathrm{vol}}\left(r\right)=\left\{\begin{array}{cc} 0 & \left(|V-V_{0}|\leq \Delta V\right)\\ \infty & \left(\mathrm{otherwise}\right) \end{array}\right.. \end{array} \end{array}$$

This Hamiltonian S(r,σ) does not include the energy term for the light irradiation. A detailed description of how to treat this important term is provided in the next section. Here, we describe the terms included in the basic Hamiltonian S(r,σ) in more detail.

The first term $S_{0}$ is the Lebwohl-Lasher potential, which is always assumed for the nematic transition of LC molecules [43]. Because of this non-polar interaction of σ in $S_{0}$, σ is identified as −σ, and $\sigma_{i}\cdot \sigma_{j}\;\to \;1$ ($\sigma_{i}\cdot \sigma_{j}\;\to \;0$) is expected for sufficiently large (small) λ.

The second term is a discrete version of the continuous $S_{1}$ in Equation (1). In the discrete $S_{1}$, $\bar{N}$ is given by

(4) $$\begin{array}{c} \bar{N}=\left(1/N_{B}\right)\sum_{ij}n_{ij}, \end{array}$$

where $n_{ij}$ denotes the total number of tetrahedra sharing the bond ij, and $N_{B}\left(=\;\sum_{ij}1\right)$ is the total number of bonds. The symbol $\sum_{ij}$ in $S_{1}$ denotes the sum over all bonds ij, “tet” in $\gamma_{ij}\left(\mathrm{tet}\right)$ denotes all tetrahedra connected to the bond ij, and $\gamma_{ij}\left(\mathrm{tet}\right)$ for the tetrahedron in Figure 3a are given by

(5) $$\begin{array}{c} \begin{array}{c} \gamma_{12}=\frac{v_{12}}{v_{13}v_{14}}+\frac{v_{21}}{v_{23}v_{24}},\;\gamma_{13}=\frac{v_{13}}{v_{12}v_{14}}+\frac{v_{31}}{v_{32}v_{34}},\;\gamma_{14}=\frac{v_{14}}{v_{12}v_{13}}+\frac{v_{41}}{v_{43}v_{42}},\\ \gamma_{23}=\frac{v_{23}}{v_{21}v_{24}}+\frac{v_{32}}{v_{31}v_{34}},\;\gamma_{24}=\frac{v_{24}}{v_{23}v_{21}}+\frac{v_{42}}{v_{41}v_{43}},\;\gamma_{34}=\frac{v_{34}}{v_{31}v_{32}}+\frac{v_{43}}{v_{41}v_{42}}. \end{array} \end{array}$$

We should note that $\gamma_{ij}=\gamma_{ji}$ and, hence, $\Gamma_{ij}=\Gamma_{ji}$ for all ij.

$\kappa S_{2}$ is an energy term that controls the stiffness of the material, and the coefficient κ is the stiffness constant. In $S_{2}$, $\sum_{i}$ denotes the sum of internal angles *i* of all triangles, and $\phi_{i}$ is the internal angle as shown in Figure 3a. For sufficiently large κ, all internal angles of triangles are expected to be $\phi_{i}\to \pi /3$, and hence the shape of tetrahedra becomes regular. In contrast, for sufficiently small κ, the tetrahedra can considerably deviate from the regular shape.

The term $U_{3D}\left(r\right)$ is the constraint potential for maintaining the positivity of each tetrahedron volume. The final term $U_{\mathrm{vol}}\left(V_{0}\right)$ is also the constraint potential for maintaining a constant total volume, which is given by $V_{0}$. This $V_{0}$ is determined from the model without $U_{\mathrm{vol}}\left(V_{0}\right)$. Details of this point are discussed in the next section.

The discrete partition function is given by

(6) $$\begin{array}{c} Z=\sum_{\sigma }\int \prod_{i=1}^{N}dr_{i}\exp\left[-S\left(r,\sigma ;U_{\mathrm{vol}}\right)\right], \end{array}$$

where $U_{\mathrm{vol}}$ in $S(r,\sigma ;U_{\mathrm{vol}})$ is written to highlight the constraint on the volume. $\sum_{\sigma }$ denotes the sum over all possible values of $\sigma (=\left\{\sigma_{1},\sigma_{2},\cdots ,\sigma_{N}\right\})$, and $\int \prod_{i=1}^{N}dr_{i}$ denotes the 3N-dimensional multiple integrations.

## 4. Monte Carlo Technique and Implementation of Light Irradiation Effect

The standard Metropolis technique is used to update the variables r and σ [44,45]. A new variable $r^{\prime }=r+\delta r$ is accepted with the probability Min[1,exp(−δS)], where δS=S(new)−S(old). The rate of acceptance of $r^{\prime }$ is controlled to be approximately 50% by the radius $R_{0}$ of the sphere containing the three-dimensional random vector δr. On the other hand, a new value $\sigma^{\prime }$ is randomly determined on the unit sphere $S^{2}$ using three different random numbers, and it is independent of the old value σ. To be more precise, three different uniform random numbers $\sigma_{x,y,z}\in \left(-0.5,0.5\right]$ are generated with the constraint $\sigma_{x}^{2}+\sigma_{y}^{2}+\sigma_{z}^{2}\leq 0.25$. This constraint makes the point $\left(\sigma_{x},\sigma_{y},\sigma_{z}\right)$ uniform in the sphere of radius 0.5. Then, the vector $\left(\sigma_{x},\sigma_{y},\sigma_{z}\right)$ is normalized such that $\sigma_{x}^{2}+\sigma_{y}^{2}+\sigma_{z}^{2}=1$. Thus, the distribution of this unit vector $\left(\sigma_{x},\sigma_{y},\sigma_{z}\right)$ is expected to be uniform on the unit sphere.

We should note that S(r,σ) is invariant under σ→−σ transformation, i.e., that σ is degenerate, because of the non-polar interaction in $S_{0}$, as mentioned above. The rate of acceptance of $\sigma^{\prime }$ is uncontrollable and depends on the coefficient λ of the energy $S_{0}$ in Equation (3). The total number of iterations called Monte Carlo sweeps (MCS), is typically $5\times 10^{7}$ to $1\times 10^{8}$ after $5\times 10^{6}$ thermalization MCS.

We now describe how to implement “the light irradiation” in the model. As described in the Introduction and in Section 2, the effect of light irradiation is to reduce the orientation ordering of σ [23,24,25,26,27,28]. To focus on this effect, we should remind ourselves that the ordering of σ can be controlled by the parameter λ in the FG model. Indeed, for a sufficiently large λ, the variables σ align spontaneously into a single direction, and the system becomes nematic if no external force is applied; on the contrary, for a sufficiently small λ, σ becomes isotropic under the same conditions for the external forces. Another technique for the implementation of this effect is to introduce temperature into the model as 1/T directly in the Boltzmann factor exp(−S/T) in Equation (6). However, this factor 1/T changes all of the coefficients λ, γ(=1) and κ to λ/T, γ/T and κ/T, respectively, where γ is the surface tension coefficient and is not included in *S* of Equation (3). If we assume a sufficiently high temperature such as T→∞, then the new factors become γ/T→0 and κ/T→0. However, this modification is expected to be too strong for the model because γ/T→0 can make the tetrahedron infinitely oblong under the constraint $U_{\mathrm{vol}}$. Therefore, it is better to use λ to change the orientation of σ.

**Figure 4:**
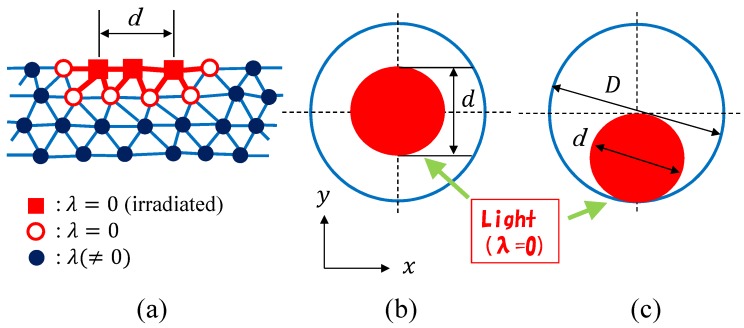
(**a**) An illustration of irradiated vertices, their neighboring vertices and non-irradiated vertices at a section of the disk lattice, (**b**,**c**) top view of irradiated disks, where the ratio d/D=0.5 and the center of irradiated domain in (**b**) are different from those in (**c**).

The problem is how to modify λ to implement the effect of light irradiation. Recalling that the interaction ${\left(\sigma_{i}\cdot \sigma_{j}\right)}^{2}$ in $S_{0}$ is defined on the bond ij, we find that one possible modification of the model is to change the first term $S_{0}$ in *S* of Equation (3) as follows:

(7) $$\begin{array}{c} S_{0}^{\prime }=\frac{1}{2}\sum_{ij}\lambda_{ij}\left(1-3{\left(\sigma_{i}\cdot \sigma_{j}\right)}^{2}\right),\;\lambda_{ij}=\left\{\begin{array}{cc} 0 & \left(\mathrm{vertex}\;i\;\mathrm{or}\;j\;\mathrm{is}\;\mathrm{irradiated}\right)\\ 1 & \left(\mathrm{otherwise}\right) \end{array}\right., \end{array}$$

where the irradiated vertex *i* or *j* is on the upper surface of the disk. In this definition, the irradiation of bond ij is represented by λ=0 on this bond. Indeed, it is easy to see that $\lambda S_{0}^{\prime }$ for $\lambda_{ij}=0$ (⇔ bond ij is irradiated) is identical to $\lambda S_{0}$ for λ=0, which corresponds to the disordered phase of σ, and that $\lambda S_{0}^{\prime }$ is identical to $\lambda S_{0}$ in the case of $\lambda_{ij}=1$ (⇔ bond ij is not irradiated). In Figure 4a, we illustrate the irradiated (⇔ solid square symbol) and not irradiated (⇔ open and solid circles) vertices. The vertices inside the domain with diameter *d* are irradiated. The vertices that are not irradiated are divided into two groups: one contains the vertices that are connected to the irradiated vertices (⇔ open circle), and the other contains the vertices that are not directly connected to the irradiated vertices (⇔ solid circle). The domains for light irradiation are shown in Figure 4b,c, where the alignment of the nematic director is in the *x*-direction.

If we consider 1/λ to be the temperature, the definition of the light irradiation in Equation (7) can be rephrased as follows: the temperature is defined on the bonds through $\lambda_{ij}$ in Equation (7) such that $\lambda_{ij}=0$ ($\lambda_{ij}=1$) corresponds to a high (low) temperature. This implies that only bond ij, one of the vertices of *i* and *j* is irradiated at least, corresponds to the high temperature (see Figure 4a) and that all of the remaining bonds correspond to the low temperature characterized by 1/λ. Please note that $S_{0}^{\prime }$ is identical to $S_{0}$ in Equation (3) if R=0. Indeed, if R=0, then all vertices are not irradiated, and hence, $\lambda_{ij}=1$ for all bonds ij. To summarize, the introduction of $\lambda_{ij}$ in Equation (7) is equivalent to introduce a domain of disordered nematic directors.

We should note that the irradiated vertices inside the region of radius *d* fluctuate around their original positions and remain irradiated, even when they move outside the region. This point that the irradiated vertices are fixed and independent of their positions is different from the experimental situation, where the irradiated position on the surface is not always identical to the positions of molecules, which also fluctuate thermally. However, our strategy is not so poor because in real LCE samples, the total number of irradiated LC molecules inside the irradiated region is sufficiently large compared to that of the boundary. The light intensity is not always uniform, and the intensity on the boundary is weaker than that of the center of light spot. For these reasons, the modeling for the light irradiation in the FG model in Equation (7) is sufficiently simple and is considered a good approximation for the experiment.

We comment on how to obtain the constant volume $V_{0}$ for the constraint $U_{\mathrm{vol}}\left(V_{0}\right)$ in Equation (3). Here, we call the simulation with (without) $U_{\mathrm{vol}}\left(V_{0}\right)$ a *V*-fix (*V*-free) simulation. To obtain $V_{0}$, we perform *V*-free simulations under the same parameters κ and λ as in the *V*-fix simulations to be performed. In this *V*-free simulation, the light is not irradiated (⇔d/D=0) because the light irradiation does not change the volume of the sample LCE in the experiment [1].

## 5. Simulation Results

### 5.1. Simulation Unit and Physical Unit

Before presenting the simulation data, we comment on the simulation/physical unit, especially the length unit, although the simulation results are not compared to the experimental data, except for the shape of the LCE disks. In the simulations, $k_{B}T$, which has the unit Nm, is always fixed to 1, where $k_{B}$ and *T* are the Boltzmann constant and the temperature, respectively. Another parameter that is modified for simplicity is the lattice spacing *a* m, which is also fixed to a=1. These parameters are eliminated from the simulation data for simplicity. Therefore, all quantities with units of length should be multiplied by *a* if these are compared to the corresponding experimental data. The problem is whether the physical value of *a* is meaningful. If the physical length of *a* is less than the order of distance between LC molecules such as 10 $D_{\mathrm{vdw}}$, where $D_{\mathrm{vdw}}$ is the van der Waals distance, it is very hard to interpret the simulation results. Therefore, we check this point here.

The problem is that we have no physical quantity that can be compared with the experimental data, except for the shapes of the LCE disk, which are obtained as snapshots. One possible value for *a* is $a=1\times 10^{-7}$ m, which was obtained in Appendix B of Ref. [21]. This value can be used for the model in this paper because the models in this paper and in Ref. [21] are the same, except for the following two facts. First, in Ref. [21], a mechanical constraint is imposed on the LCE to calculate the stress strain diagram, while it is not imposed on the model in this paper; second, the constraint for the volume $U_{\mathrm{vol}}\left(r\right)$ is imposed on the model in this paper, but it is not imposed on the model in Ref. [21]. Another possible value for *a* can be obtained by comparing the disk size. The experimental disk size is $D_{\exp}=5$ mm, as mentioned in Section 2, while it is approximately given by D≃10 in the simulation units, even though this *D* is slightly dependent on the simulation conditions, such as λ. To compare this *D* with $D_{\exp}$, we should multiply *D* by the constant *a* to obtain $D_{\exp}=aD$; we have

(8) $$\begin{array}{c} a=D_{\exp}/D=5\times 10^{-4}\;m. \end{array}$$

This value is relatively larger than the value above $a=1\times 10^{-7}$ m; however, it is sufficient because σ is a coarse-grained variable. To be more precise, the variable σ should be understood as an average direction of many LC molecules, or in other words a group of LC molecules is represented by a single σ. This coarse-graining is not special to the FG model but it is always assumed in lattice simulations for spin models for example.

Using $D_{\exp}=aD$, D=10 and $a≃10^{-9}\;m$, which is approximately 10 times larger than $D_{\mathrm{vdw}}$, we have the minimum size $D_{\exp}=10^{-8}\;m$, to which the model is applied. This size is sufficiently small for technological applications such as actuators. In contrast, we have no limitation on the maximum size of $D_{\exp}$. Therefore, we can actually assume arbitrary size for the disk, because the lattice spacing *a* is an arbitrary parameter except for the constraint $a>10^{-9}\;m$.

### 5.2. Snapshots

**Figure 5:**
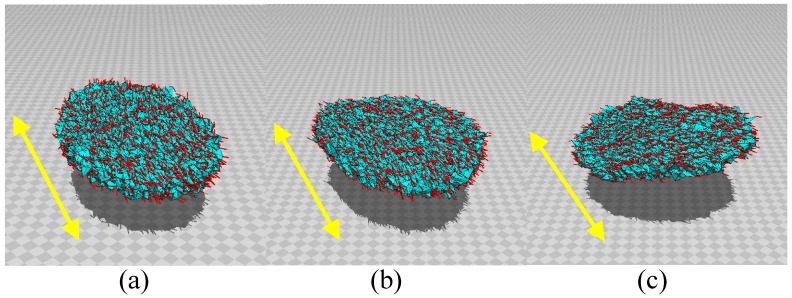
Snapshots of disks obtained in the simulations without light irradiation and under the conditions (**a**) λ=1.2, (**b**) λ=2.4, and (**c**) λ=1.2 with κ=0.5. The sizes are (**a**) D/H=12, N= 10,566, (**b**) D/H=16, N= 10,980, and (**c**) D/H=20, N= 10,563. The arrows (↔) represent the nematic director direction.

Figure 5a–c are snapshots of disks without light irradiation (⇔d/D=0), and the sizes of the lattices are (a) D/H=12, N= 10,566, (b) D/H=16, N= 10,980, and (c) D/H=20, N= 10,563. Note that these D/H values of the original disks are different from the actual D/H values of the lattices in Figure 5a–c: these lattices are anisotropically deformed because the system is in the nematic phase, as discussed below. The parameter κ is assumed to be κ=0.5 for all disks. We also performed simulations for κ=0 and κ=0.8; however, these are not suitable for our purpose. In fact, the disks are not always flat for κ=0, even in the absence of light irradiation, because of the large shape fluctuations. By contrast, the bending of the disks is not always sufficient for κ=0.8, even when the light is irradiated. Therefore, we consider that κ=0 is too small and that κ=0.8 is too large. Therefore, we assume that κ=0.5.

The parameter λ should be sufficiently large because the LCE sample is in the nematic phase on the water; therefore, we assume (a) λ=1.2, (b) λ=2.4, and (c) λ=1.2 to realize the nematic configurations. We find in Figure 5a–c that the directors σ align in specific directions, as indicated by the arrows on the figures. These directions are determined by the initial configuration for σ in MC simulations because the assumed λs are sufficiently large for the system to remain in the initial nematic configuration. As mentioned above, the disks deform their shape from the original circle shape to the ellipse shape, in which the diameter along the director direction is slightly larger than the diameter along the perpendicular direction. Moreover, the thickness *H* of the disks shrinks from their initial values, corresponding to the initial lattices in Figure 2a–c. If λ is too small, as in the case of λ=0, the director σ becomes random and does not align, even when the initial value of σ is fixed to a nematic configuration.

We should emphasize that the director σ becomes random in the isotropic phase if λ is fixed to λ=0, for example, because the tetrahedra of the 3D disks are relatively close to the regular shape in their original configurations, as shown in Figure 2a–c. For these configurations, there is no reason for σ to align to a direction even when this direction is given as an initial configuration. On the other hand, if the configuration of σ is given by an initially aligned direction at sufficiently large λ, this makes the shape of the tetrahedra deformed or anisotropic. Therefore, the disks shown in Figure 5a–c obtained at sufficiently large λ are understood to be “stressed” by the alignment of σ, even though these are in the minimum energy states.

**Figure 6:**
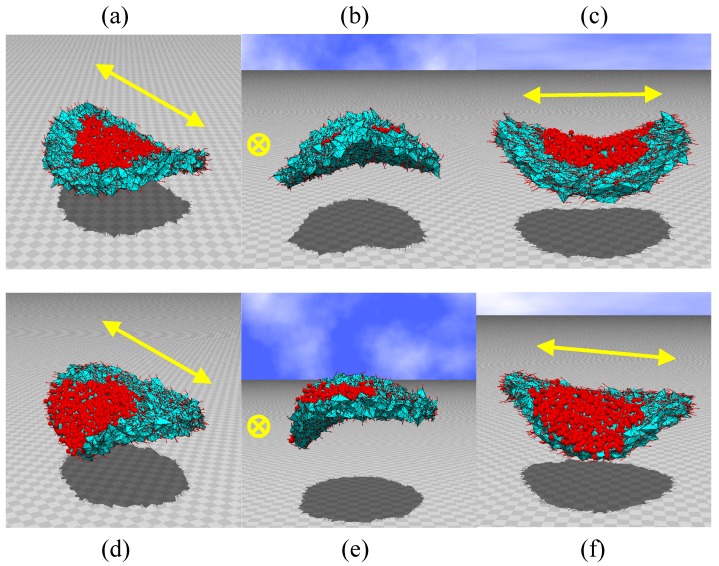
(**a**–**c**) Snapshots of disks irradiated by light at the central region, and (**d**–**f**) snapshots of disks irradiated at the region between the center and edge. The small spheres represent the irradiated vertices and their nearest neighbor vertices, and the symbols (↔) and (⊗) represent the nematic director directions. The original disk size is D/H=12, which corresponds to the size in Figure 1a. It is assumed in the simulations that κ=0.5 and λ=1.2. N= 10,566.

We show the snapshots of bending disks irradiated at the central part (Figure 6a–c) and those irradiated at one side between the center and edge (Figure 6d–f), which correspond to Figure 4b,c, respectively. The size of the disk is D/H=12, N= 10,566, which corresponds to the disk shown in Figure 2a and Figure 5a. Small spheres on the upper surface of the snapshots in Figure 6 denote the irradiated vertices and the vertices connected to the irradiated vertices (see Figure 4a). The arrows (↔) and the symbol (⊗) denote the direction of the directors σ, which are shown in the snapshots by the small bars. We should note that the directions of almost all bars are identical to the directions indicated by (↔) and (⊗). The viewing angle of Figure 4b is vertical to that of Figure 4c, and those of Figure 4e,f are vertical to each other. From these snapshots, we find that the shape of the disks is almost the same as that of the experimentally observed ones in Figure 1a,b. Indeed, it is easy to see that the bending part of the edges moves from the center to the direction of the motion of the irradiated region.

The FG model obtains the same shape as experiment because the directors at the irradiated region become randomly oriented or form an isotropic configuration. In the isotropic phase, the 3D disk is restored to its original shape and consists of almost regular tetrahedra, and the direction of the edges of the tetrahedra becomes random. In short, the shape of the tetrahedra changes from oblong to regular as a consequence of the light irradiation. Indeed, the triangles on the upper surface remain oblong along the nematic director direction if it is not irradiated, but under light irradiation, the triangle shape changes from oblong to regular. Therefore, the irradiated region, e.g., in Figure 6a, shrinks along the direction (↔, ⊗) and expands along the direction vertical to the direction (↔, ⊗).

**Figure 7:**
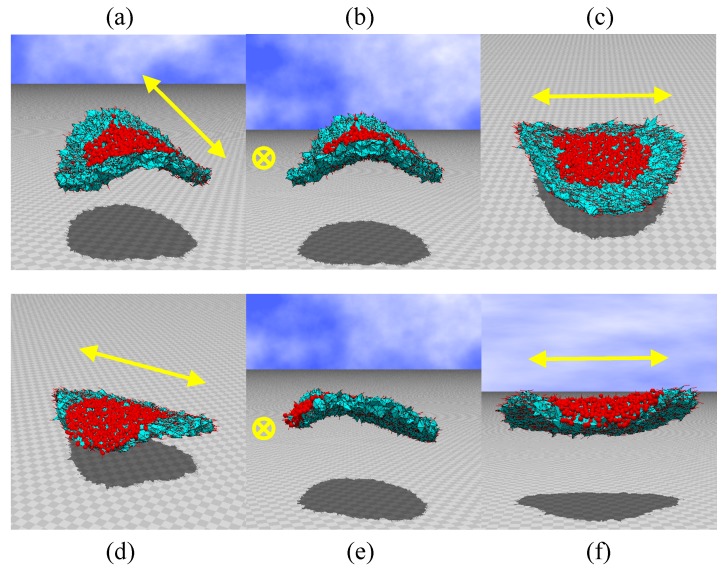
(**a**–**c**) Snapshots of disks irradiated by light at the central region, and (**d**–**f**) those irradiated at the region between the center and edge. The original disk size is D/H=16 (see Figure 2b). The parameters are κ=0.5 and λ=2.4. N= 10,980.

**Figure 8:**
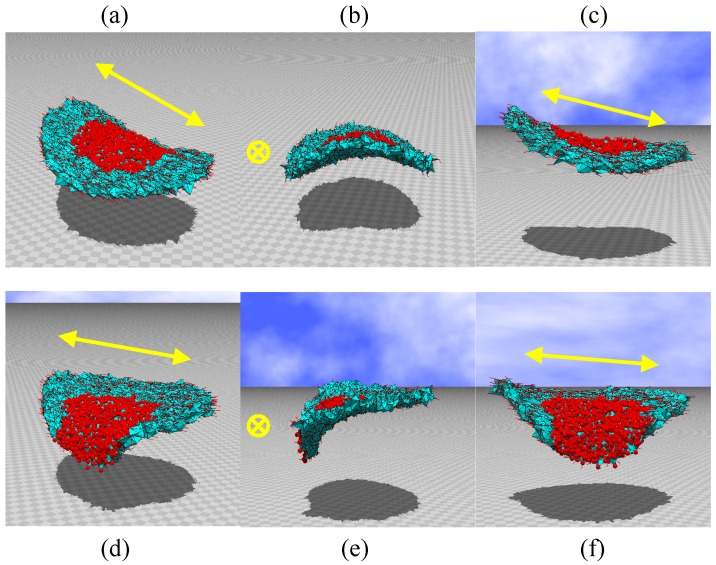
(**a**–**c**) Snapshots of disks irradiated by light at the central region, and (**d**–**f**) those irradiated at the region between the center and edge. The original disk size is D/H=20 (see Figure 2c). The parameters are κ=0.5 and λ=1.2. N= 10,563.

The simulations are performed on disks of size D/H=16, N= 10,980, and D/H=20, N= 10,563, as shown in Figure 2b,c, respectively. The snapshots are shown in Figure 7a–f and Figure 8a–f. We find that the bending shapes are almost identical to those in Figure 6a–f. This implies that the shape deformation observed in the FG model is stable in that the shape remains unchanged with respect to a small variation in disk size, which is characterized by the ratio D/H.

### 5.3. Gaussian Curvature

In this section, we show the Gaussian curvature, which characterizes the shape of the disks bending similar to the disks shown in the previous section [40]. This quantity cannot always be compared to experimental data because no experimental data have been reported. However, a quantitative characterization of the bending by light irradiation is interesting, and the Gaussian curvature reflects surface bending. For these reasons, we calculate the curvature energy $S_{G}$ on the upper surface, where the light is illuminated, and on the lower surface in contact with water, and we examine the difference between these $S_{G}$ values.

**Figure 9:**
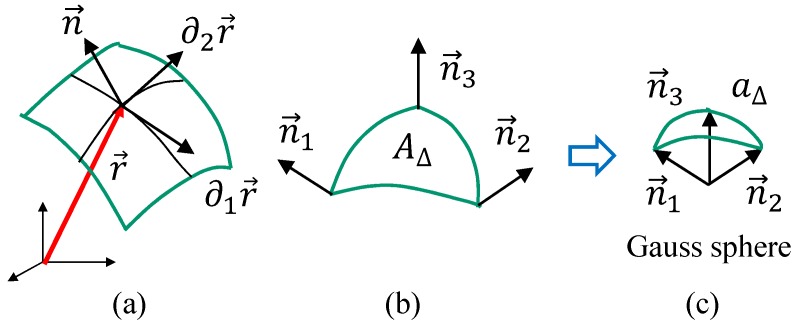
(**a**) Tangential vectors $\partial_{1}r$ and $\partial_{2}r$, and the corresponding unit normal vector n on a smooth surface in ${ℜ}^{3}$, (**b**) a smooth triangle of area $A_{\Delta }$ and with the normal vectors $n_{i}\left(i=\;1,2,3\right)$ at the vertices, (**c**) a part of the Gauss sphere of area $a_{\Delta }$ corresponding to the triangle in (**b**).

The absolute Gaussian curvature |K| is defined by $\left|K|=\right|\partial_{1}n\times \partial_{2}n|/|\partial_{1}r\times \partial_{2}r|$ [40], where $\partial_{i}r$ is a tangential vector along the local coordinate axis $x_{i}\left(i=1,2\right)$, and n is a unit tangential vector (see Figure 9a). Since $|\partial_{1}n\times \partial_{2}n|/|\partial_{1}r\times \partial_{2}r|$ and $\int \sqrt{g}d^{2}x$ can also be written as

(9) $$\begin{array}{c} \frac{\left|\partial_{1}n\times \partial_{2}n\right|}{\left|\partial_{1}r\times \partial_{2}r\right|}=\lim_{A_{\Delta }\to 0}\frac{a_{\Delta }}{A_{\Delta }},\;\int \sqrt{g}d^{2}x=\int dA, \end{array}$$

we have

(10) $$\begin{array}{c} \int \sqrt{g}d^{2}x\left|K\right|=\int dA\lim_{A_{\Delta }\to 0}\frac{a_{\Delta }}{A_{\Delta }}. \end{array}$$

On the right-hand side of the first term of Equation (9), $A_{\Delta }$ denotes the area of a smooth triangle, as shown in Figure 9b, and $a_{\Delta }$ is the corresponding area on the Gauss sphere (see Figure 9c). Recalling that ∫dA can be replaced by $\sum_{\Delta }A_{\Delta }$ in the limit of $A_{\Delta }\to 0$, we have a discrete version of the absolute Gaussian curvature, which we write as $S_{G}$ such that

(11) $$\begin{array}{c} S_{G}=\sum_{\Delta }a_{\Delta }. \end{array}$$

This $S_{G}$ can be calculated on the upper and lower surfaces of the 3D disks because these surfaces are considered as triangulated 2D disks. For each triangle Δ, $a_{\Delta }$ is calculated using the three unit normal vectors at the three vertices (Figure 9c). The unit normal vector $n_{i}$ at the vertex *i* is defined by

(12) $$\begin{array}{c} n_{i}=\frac{\sum_{j(i)}A_{j(i)}N_{j(i)}}{\left|\sum_{j(i)}A_{j(i)}N_{j(i)}\right|}, \end{array}$$

where $N_{j(i)}$ is the unit normal vector of triangle j(i) connected to vertex *i* and $A_{j(i)}$ is its area.

**Figure 10:**
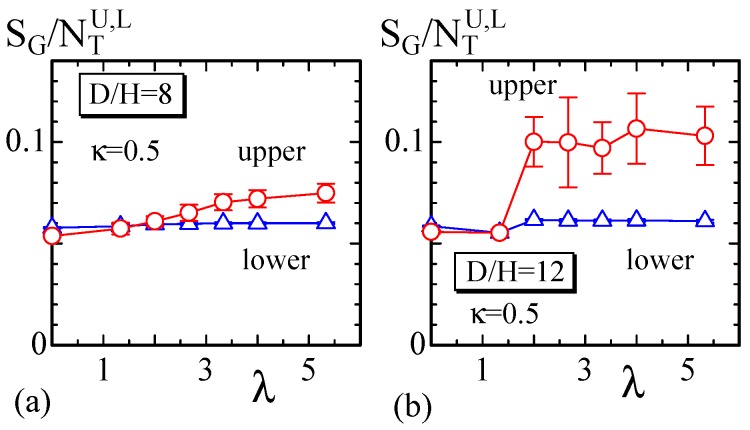
$S_{G}/N_{T}^{U,L}$ vs. λ obtained on the disks of (**a**) D/H=8 and (**b**) D/H=12. The model for this calculation has no constraint $U_{\mathrm{vol}}$.

First, we plot $S_{G}/N_{T}^{U,L}$ vs. λ of the model without the constraint $U_{\mathrm{vol}}$ in Figure 10a,b to examine how $S_{G}$ reflects the effect of light irradiation on the upper surface. The size of the disk used for the results in Figure 10a is D/H=8 and N= 10,346, which is relatively thick, whereas for Figure 10b, D/H=12 and N= 10,566, as shown in Figure 2a. The light is irradiated on all of the vertices of the upper side of the disks, and this condition is expressed by d/D=1 in Figure 4b. The symbols $N_{T}^{U}$ and $N_{T}^{L}$ denote the total number of triangles on the upper and lower surfaces, respectively (Table 1). We find that there is a nontrivial difference between $S_{G}/N_{T}^{U}$ and $S_{G}/N_{T}^{L}$, which are $S_{G}$ per triangle on the upper and lower surfaces, respectively, for a large λ region. For the disk with D/H=8, the difference is relatively small, even when λ is sufficiently large. By contrast, the difference can be clearly seen for the disk with D/H=12 in Figure 10b.

**Figure 11:**
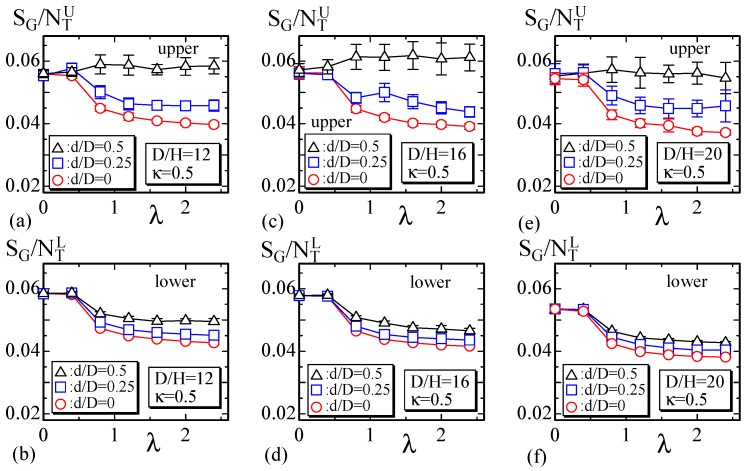
$S_{G}/N_{T}^{U,L}$ vs. λ obtained for the disk with size (**a**,**b**) D/H=12, (**c**,**d**) D/H=16, and (**e**,**f**) D/H=20. The data $S_{G}/N_{T}^{U}$ (**a**,**c**,**e**) for the upper surface depend on d/D, and for the lower surface, only a slight dependence can be seen.

Next, we show the results obtained for the disks whose snapshots are shown in Section 5.2, where the constraint $U_{\mathrm{vol}}$ is imposed. The results of the lattice of size D/H=12, D/H=16, and D/H=20, are plotted in Figure 11a,b, Figure 11c,d, and Figure 11e,f, respectively. On the upper (lower) Figure 11a,c,e, (Figure 11b,d,f), the results $S_{G}/N_{T}^{U}$ ($S_{G}/N_{T}^{L}$) calculated on the upper (lower) surface are plotted. The results at λ=0.5 (and at λ=0) are independent of the size of light spot d/D and are almost independent of whether the surface is upper or lower. This is because d/D=0 on the light spot has no non-trivial influence on the ordering of σ, which is actually not ordered under λ≤0.5 in the whole other region. By contrast, $S_{G}/N_{T}^{U}$ on the upper surface becomes dependent on the spot size d/D for the large λ region. This occurs because the light irradiation defined by λ=0 on the region of diameter *d* destroys the nematic order of σ for a large λ. We should note that $S_{G}/N_{T}^{L}$ depends on d/D, even on the lower surface, where no light is illuminated. For the large λ region, we find that $S_{G}/N_{T}^{L}$ at d/D=0.5 is non-trivially larger than $S_{G}/N_{T}^{L}$ at d/D=0. This non-trivial difference indicates that the disordered configuration of σ on the upper surface for d/D=0.5 influences σ on the lower surface.

## 6. Summary and Conclusions

We used MC simulations to demonstrate that the Finsler geometry (FG) model successfully reproduces the experimental results of LCE shape deformation under light irradiation reported in [1]. In the simulations, the irradiated region is given by a circle on the disk, and the center of the irradiated circle is located at the center of the disk and at the midpoint of the center and edge of the disk. For a sufficiently large λ, which is the coefficient of Lebwohl-Lasher potential $S_{0}$, the bending shape of the disk is almost identical to the experimentally observed shapes reported in [1].

For the FG model, the reason for the bending is simply understood as the orientation σ becoming disordered (⇔ isotropic) on the irradiated region but ordered (⇔ nematic) on the other parts of the LCE disk. This change in the orientation order of σ comes from the variation of λ. The role of λ is simply to make the orientation of σ ordered (disordered) if it is sufficiently large (small). On the other hand, in the FG model itself, the ordering of σ and the macroscopic shape are connected by the interaction between the direction σ of director and the position r of polymer. This interaction is indirectly introduced by a modification of the intrinsic geometry of materials from Euclidian to Finsler. Using the interaction introduced by the FG model technique, we find that (i) the shape of disk is determined only by the change in the director orientation and (ii) the obtained results are consistent with the experimental observations.

We should emphasize that in FG modeling, it is not necessary to delve into the details of the temperature and photoisomerization effects. In this sense, the FG model is a coarse-grained model and, hence, can be applied to many phenomena that are not always elucidated from the microscopic perspective. There is no limitation on the size of sample to which FG modeling is applied, although the sample LCE size targeted in this paper is limited in the range of a few mm.

## Figures and Tables

**Table 1:** The four different lattices used in this paper. The first lattice of size N= 10,346 is used only for the calculation of Gaussian curvature.

*N*	$N_{B}$	$N_{T}$	$N_{\mathrm{tet}}$	D/H	$N^{U,L}$	$N_{B}^{U,L}$	$N_{T}^{U,L}$
10,346	69,964	116,041	56,422	8	1344	3916	2573
10,566	70,503	116,037	56,099	12	1718	5023	3306
10,980	72,049	117,623	56,553	16	2088	6120	4033
10,563	67,752	109,460	52,270	20	2322	6814	4493

## Abbreviations

The following abbreviations are used in this manuscript:

LCE	Liquid crystal elastomer
LC	Liquid crystal
FG	Finsler geometry
MC	Monte Carlo
MCS	Monte Carlo sweep

## References

[1] Chamacho-Lopez M., Finkelmann H., Palffy-Muhoray P., Michael S. (2004). Fast liquid-crystal elastomer swims into the dark. Nat. Mater..

[2] Warner M., Terentjev E.M. (2007). Liquid Crystal Elastomer.

[3] Donald A.M., Windle A.H., Hanna S. (2006). Liquid Crystalline Polymers.

[4] Domenici V. (2012). ^2^H NMR studies of liquid crystal elastomers: Macroscopic vs. molecular properties. Prog. Nucl. Mag. Res. Spectrosc..

[5] Terentjev E.M. (1999). Liquid crystalline networks. Curr. Opin. Colloid Interface Sci..

[6] Yu Y., Nakano M., Ikeda T. (2004). Photoinduced bending and unbending behavior of liquid-crystalline gels and elastomers. Pure Appl. Chem..

[7] Ikeda T., Ube T. (2011). Photomobile polymer materials: From nano to macro. Materialstoday.

[8] Urayama K., Kondo H., Arai Y.O., Takigawa T. (2005). Electrically driven deformations of nematic gels. Phys. Rev. E.

[9] Urayama K., Honda S., Takigawa T. (2005). Electrooptical Effects with Anisotropic Deformation in Nematic Gels. Macromolecules.

[10] Urayama K., Honda S., Takigawa T. (2006). Deformation of Coupled to Director Rotation in Swollen Nematic Elastomers under Electric Fields. Macromolecules.

[11] Yusril Y., Huh J.H., Cladis P.E., Brand H.R., Finkelman H. (2005). Low-voltage-driven electromechanical effects of swollen liquid-crystal elastomers. Phys. Rev. E.

[12] Nishikawa E., Finkelman H. (1999). Smectic—A liquid single crystal elastomers–strain induced break-down of smectic layers. Macromol. Chem. Phys..

[13] Shahinpoor M., Kim K.J., Mojarrad M. (2007). Artificial Muscles: Applications of Advanced Polymeric Nanocomposites.

[14] Stefano P., Andrew G.M., Shang Y.R., Kai K.M., Tian Q., Hao Z., Camilla P., Daniele M., Alberto S.-C., Nadia K. (2016). Structured light enables biomimetic swimming and versatile locomotion of photoresponsive soft microrobots. Nat. Mater..

[15] Finkelmann H., Kim S.T., Munoz A., Palffy-Muhoray P., Taheri B. (2001). Tunable Mirrorless Lasing in Cholesteric Liquid Crystalline Elastomers. Adv. Mater..

[16] Resetic A., Milavec J., Zupancic B., Domenici V., Zalar B. (2016). Polymer-dispersed liquid crystal elastomers. Nat. Commun..

[17] Geng Y., Almeida P.L., Femandes S.N., Cheng C., Palffy-Muhoray P., Godinho M.H. (2013). A cellulose liquid crystal motor: a steam engine of the second kind. Sci. Rep..

[18] Mbanga B.L., Ye F., Selinger J.V., Selinger R.L.B. (2010). Modeling elastic instabilities in nematic elastomers. Phys. Rev. E.

[19] Wei Z., Shelley M., Palffy-Muhoray P. (2011). Modeling and simulation of liquid-crystal elastomers. Phys. Rev. E.

[20] Koibuchi H., Sekino H. (2014). Monte Carlo studies of a Finsler geometric surface model. Physica A.

[21] Osari K., Koibuchi H., Sekino H. (2017). Finsler geometry modeling and Monte Carlo study of 3D liquid crystal elastomer. Polymer.

[22] Takano Y., Koibuchi H. (2017). J-shaped stress-strain diagram of collagen fibers: Frame tension of triangulated surfaces with fixed boundaries. Phys. Rev. E.

[23] Finkelmann H., Nishikawa E. (2001). A New Opto-Mechanical Effect in Solids. Phys. Rev. Lett..

[24] Sanchez-Ferrer A., Finkelmann H. (2010). Thermal and mechanical properties of new Main-Chain Liquid-Crystalline Elastomers. Solid State Sci..

[25] Jin L., Yan Y., Huo Y. (2010). A gradient model of light-induced bending in photochromic liquid crystal elastomer and its nonlinear behaviors. Int. J. Non-Linear Mech..

[26] Dunn M.L., Maute K. (2009). Photomechanics of blanket and patterned liquid crystal elastomer films. Mech. Mater..

[27] Knezevic M., Warner M., Copic M., Sanchez-Ferrer A. (2013). Photodynamics of stress in clamped nematic elastomers. Phys. Rev. E.

[28] Warner M., Mahadevan L. (2004). Photoinduced Deformations of Beams, Plates, and Films. Phys. Rev. Lett..

[29] Baer E., Hiltner A., Keith H.D. (1987). Hierarchical structure in polymeric materials. Science.

[30] Lakes R. (1993). Materials with structural hierarchy. Nature.

[31] Lin Y.-C., Chen H.-L., Hashimot O.T., Chen S.-A. (2016). Mechanism of Hierarchical Structure Formation of Polymer/Nanoparticle Hybrids. Macromolecules.

[32] David F., Nelson D., Piran T., Weinberg S. (2004). Geometry and field theory of random surfaces and membranes. Statistical Mechanics of Membranes and Surfaces.

[33] Gompper G., Kroll D.M., Nelson D., Piran T., Weinberg S. (2004). Triangulated-surface models of fluctuating membranes. Statistical Mechanics of Membranes and Surfaces.

[34] Kantor Y., Nelson D.R. (1987). Phase transitions in flexible polymeric surfaces. Phys. Rev. A.

[35] Wheater J.F. (1994). Random surfaces: From polymer membranes to strings. J. Phys. A Math. Gen..

[36] Kownacki J.-P., Diep H.T. (2002). First-order transition of tethered membranes in three-dimensional space. Phys. Rev. E.

[37] Essafi K., Kownacki J.-P., Mouhanna D. (2014). First-order phase transitions in polymerized phantom membranes. Phys. Rev. E.

[38] Cuerno R., Caballero R.G., Gordillo-Guerrero A., Monroy P., Ruiz-Lorenzo J.J. (2016). Universal behavior of crystalline membranes: crumpling transition and Poisson ratio of the flat phase. Phys. Rev. E.

[39] Doi M., Edwards S.F. (1986). The Theory of Polymer Dynamics.

[40] Matsumoto M. (1975). Keiryou Bibun Kikagaku (in Japanese).

[41] Matsumoto M. (1986). Foundations of Finsler Geometry and Special Finsler Spaces..

[42] Bao D., Chern S.-S., Shen Z. (2000). An Introduction to Riemann-Finsler Geometry, GTM 200.

[43] Lebwohl P.A., Lasher G. (1972). Nematic-Liquid-Crystal Order—A Monte Carlo Calculation. Phys. Rev. A.

[44] Metropolis N., Rosenbluth A.W., Rosenbluth M.N., Teller A.H., Teller E. (1953). Equation of state calculations by fast computing machines. J. Chem. Phys..

[45] Landau D.P. (1976). Finite-size behavior of the Ising square lattice. Phys. Rev. B.

